# Oviposition preference of cabbage white butterflies in the framework of costs and benefits of interspecific herbivore associations

**DOI:** 10.1098/rsos.150524

**Published:** 2015-12-02

**Authors:** Kaori Shiojiri, Maurice Sabelis, Junji Takabayashi

**Affiliations:** 1 Center for Ecological Research, Kyoto University, 2-509-3 Hirano, Otsu, Shiga 520-2113, Japan; 2 The Hakubi Center for Advanced Research, Kyoto University, Yoshida Ushinomiya, Sakyo-ku, Kyoto 606-8501, Japan; 3 Institute for Biodiversity and Ecosystem Dynamics, University of Amsterdam, Kruislaan 320, 1098SM Amsterdam, The Netherlands

**Keywords:** ants, cabbage plants, oviposition decision, *Pieris rapae*, *Plutella xylostella*

## Abstract

When deciding where to oviposit, herbivorous insects consider: (i) the plant’s value as a food source, (ii) the risks of competing with con- and heterospecific herbivores, and (iii) the risks of parasitism and predation on the host plant. The presence of con- and/or heterospecific competitors would further affect the oviposition preference, because the preceding herbivores induce direct/indirect defences in plants against forthcoming herbivores, and thereby alter oviposition decisions. In previous studies, the abovementioned factors have not been studied in an integrative manner. We performed here a case study of this by assessing the oviposition preferences of a small white butterfly, *Pieris rapae*, for plants occupied by combinations of conspecific larvae, heterospecific larvae (*Plutella xylostella*), specialist parasitoids of *Pi. rapae* (*Cotesia glomerata*) and generalist predators (ants). We previously reported that the females showed equal preference for *Pl. xylostella*-infested and uninfested plants. Here, we showed that *Pi. rapae* females preferred uninfested plants to conspecific-infested ones, and *Pl. xylostella*-infested plants to *Pi. rapae*-infested ones. We discuss these oviposition preferences of *Pi. rapae* females in the framework of costs and benefits of interspecific herbivore associations from the above point of view.

## 1. Introduction

Factors determining host plant selection by herbivorous insects have been analysed by testing the relationship between measures of herbivore preference for a plant and of their reproductive performance on that plant [1–4], the effects of con- and heterospecific herbivores [5–7] and the risks of parasitism and predation on that plant [6–11]. However, one factor that has been largely overlooked is herbivore-induced indirect defence; that is, in response to herbivory, plants can promote the effectiveness of natural enemies of herbivorous insects by providing chemical information signalling the presence of herbivores on the plants [12–16]. This factor may modify a plant’s value to the herbivore and may therefore also alter the herbivore’s oviposition preference.

For herbivore-induced indirect defence by attracting natural enemies, most previous studies have focused on a defence against a single herbivore species [12–16], and some focus on the extended aspect of herbivore-induced indirect defence that emerges when plants are infested by several herbivore species at the same time [11,17–19]. Such studies show that the value of a host plant for one herbivorous species can be affected by indirect defence responses induced by other coexisting herbivores [11,17–19]. In agricultural fields, cabbage plants are frequently simultaneously infested by larvae of *Pieris rapae* (Lepidoptera: Pieridae) and *Plutella xylostella* (Lepidoptera: Plutellidae). Because caterpillars have low mobility, their mother influences their developmental success by her choice of plants on which to oviposit. We reported that *Pl. xylostella* females preferentially oviposited on plants infested by *Pi. rapae* larvae [11]. This preference is partly explained by the reduced risk of being parasitized; that is, parasitism by *Cotesia vestalis* (Hymenoptera: Braconidae), a dominant parasitoid of *Pl. xylostella* larvae, is lower on plants infested by the two species of caterpillars together than on plants infested by *Pl. xylostella* larvae alone because of the reduced attraction of *C. vestalis* to plants infested by both species [11]. Thus, the potential enemy-rare space for *Pl. xylostella* larvae was mediated by the previous infestation by *Pi. rapae* larvae. This case study shows that enemy-rare space caused by heterospecific-herbivore-induced indirect defence affects oviposition preference of other species of herbivores (herbivore-induced enemy-rare space).

We also reported that females of *Pi. rapae* show equal oviposition preference between *Pl. xylostella* larvae-infested cabbage plants and uninfested cabbage plants, and that resource competition and preference-performance do not explain this equal preference [11]. *Pieris rapae* females should oviposit eggs on uninfested plants, because cabbage plants infested by either *Pi. rapae* larvae, *Pl. xylostella* larvae or both attract *Cotesia glomerata* at the same intensity and at the same levels of damage; thereby, *Pl. xylostella* larvae-infested plants are herbivore-induced enemy-dense space. One of the objectives of this study is to try to explain this paradox. To do so, in this study, we focused on apparent interactions (a mechanism through which a species affects another species, which may or may not share resources, at the same trophic level, mediated through the action of shared natural enemies; this has been revised based on the definition of apparent competition [5,6]).

Here, we investigated factors affecting the oviposition preference of *Pi. rapae* females in habitats in which conspecific herbivores, heterospecific herbivores (*Pl. xylostella* larvae), specialist parasitoids (*C. glomerata*) and generalist predators (several ant species) are involved. By integrating potential factors affecting oviposition preference by herbivores, we show that *Pi. rapae* is tuned to a particular structure of tritrophic interaction webs in which interspecific herbivore associations are active.

## 2. Material and methods

### 2.1 Plants and insects

Cabbage plants, *Brassica oleracea* cv. Sikidori, that were more than one month old (with *ca* five leaves) were grown in a climate chamber at 25±2°C, 60% relative humidity, and 16 L:8 D fluorescent light (5500 lux). We collected adult females of *Pi. rapae* from a field near Kyoto City, Japan, and allowed them to lay eggs on these cabbage plants in the laboratory. Hatched *Pi. rapae* larvae were reared on detached cabbage leaves in the climate-controlled room (25±2°C; 50–70% RH; 16 L:8 D). *Plutella xylostella* larvae were collected from cruciferous plants in a field near Kyoto City and reared on cabbage leaves in the climate-controlled room under conditions identical to those for *Pi. rapae* larvae.

To test the oviposition preference of *Pi. rapae*, we collected adult females in the field and held them for 1 day in a cage without crucifer plants. Because *Pi. rapae* females mate soon after emergence, it was reasonable to assume that the collected females had already mated (personal communication 2000, Dr I. Kandori, Kinki University, Nara, Japan).

*Cotesia glomerata* were obtained from parasitized *Pi. rapae* larvae collected in a field near Kyoto, Japan. Adult wasps of both sexes were kept in a climate-controlled room (25±2°C, 50–70% RH, 16 L:8 D), where they were contained in a plastic cage (13×20×13 cm) with a droplet of honey as an energy source for a period of 3 days. After mating, females were transferred to a glass tube with a small amount of honey and kept in a climate-controlled room at 18±2°C, 50–70% relative humidity, and continuous darkness. They were used for experiments within 10 days of maturation. At least 1 h before the start of each experiment, oviposition-inexperienced females were transferred to another climate-controlled room (25±2°C; 50–70% relative humidity) under 16 L:8 D light conditions.

**Figure 1.**
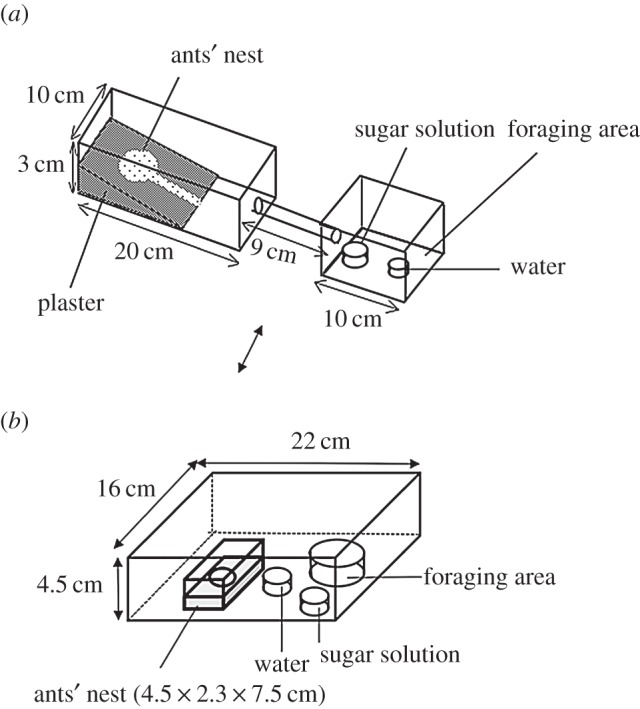
Schematic drawing of an artificial ant nest with a feeding cage that offers sugar, water and mealworms to the ants. Different nests were used (*a*) for *L. japonicus* and *P. pungens* and (*b*) for *P. flavipes* because of their differences in body size. Foraging area in (*b*) was 12 cm in diameter. Petri dishes for supplying water and sugar solution were 4 cm in diameter.

Colonies of three ant species (Hymenoptera: Formicidae: *Lasius japonicus*, *Pristomyrmex pungens* and *Paratrechina flavipes*; identified by Dr Y. Takematsu, Yamaguchi University, Yamaguchi, Japan) were sampled from a field on the campus of Kyoto University and allowed to establish new colonies in a plastic cage (10×20×3 cm) in the laboratory (figure 1*a*, *L. japonicus* and *Pr. pungens*; figure 1*b* for *Pa. flavipes* because of their body size). The ants were fed a sucrose solution and mealworms in a climate-controlled room (25±2°C, 60% relative humidity, 16 L:8 D) until use in experiments one week after founding the new colony.

### 2.2 Oviposition preference of *Pieris rapae*

Oviposition preference of *Pi. rapae* in a cage (180×180×180 cm) covered with gauze (mesh size: 1.8 mm) was assessed. The cage was positioned in an experimental field on the campus of Kyoto University. Ten mated females of *Pi. rapae* were released into the cage from 09.30 to 15.30. They were allowed to oviposit on two plants that received contrasting treatments according to one of the following two experimental designs: (i) an uninfested plant versus a plant infested by conspecific larvae, and (ii) a plant infested by conspecific larvae versus a plant infested by larvae of *Pl. xylostella*.

Visual observations in our study field showed that the naturally occurring damage levels of *Pi. rapae* larvae- or *Pl. xylostella* larvae-infested cabbage plants at the early stage were 10–30% of the leaf surface. Based on this, we used intermediate damage levels (15–20%) to test the oviposition preference. Larvae-infested plants were prepared by releasing either five second-instar *Pi. rapae* or 20 second- or third-instar *Pl. xylostella* randomly over the leaves of a plant, where they were kept for 24 h, resulting in consumption of 15–20% of the leaf surface. For both types of infested plants, all herbivores were removed at the end of the 24 h feeding period.

The experiments were performed on sunny days (20–30°C) between June and October, and each choice test was replicated three times. As eggs of *Pi. rapae* are usually laid singly on plants [20], the total number of eggs laid on the two plants represents the number of independent oviposition trials [18]. For statistical analysis, based on a preceding study [18,20], we evaluated that each egg laid was the result of an independent oviposition choice, and the data were analysed using a replicated *G*-test [21].

### 2.3 Performance of *Pieris rapae* larvae reared on uninfested plants or conspecific-infested plants

We tested the effects of the presence of a conspecific larva on the same plant on the performance of *Pi. rapae* in their larval stage. To do this, we used a first-instar *Pi. rapae* that was inoculated on a plant that had been infested 1 day earlier by one other first-instar *Pi. rapae*, or on plants that had hosted no conspecific larva. After the inoculation, the initially inoculated occupant was not removed. The initial and the later occupant were discriminable owing to their size difference and the duration of the larval period. The later occupant was reared until pupation. At this point, more than 60% of the leaf surface of a plant was consumed. The experiment was replicated on 20 plants for each treatment in a climate-controlled room (25±2°C, 50–70% relative humidity, 16 L:8 D). We compared pupal weight and duration of the larval period between uninfested plants and plants infested by a conspecific larva using a Student’s *t*-test for comparison of means. We also compared the survival rates by Fisher’s exact test.

### 2.4 Flight responses of the parasitoid *Cotesia glomerata* to *Pieris rapae*-infested plants

As mentioned above, the naturally occurring damage levels of *Pi. rapae* larvae- or *Pl. xylostella* larvae-infested cabbage plants of early stage were 10–30% of the leaf surface under field conditions. Thus, we compared 10%- and 30%-damaged plants. Larvae-infested cabbage plants were prepared as follows. Three second- or third-instar *Pi. rapae* were released randomly on the plant and allowed to feed there for 24 h. After 24 h, *ca* 10% of the leaf surface of the plant had been consumed. We then removed the larvae and their associated products (e.g. faeces, silk) from the leaf with a small, fine brush. To obtain cabbage plants with 30% damage, we released 10 instead of three second- or third-instar *Pi. rapae*.

The flight responses of females of *C. glomerata* to *Pi. rapae*-infested plants with 10% and 30% damage were observed in a cage (25×35×30 cm) with three windows covered by nylon gauze provided with a single door. The cages were placed together in a climate-controlled room (25±2°C; 50–70% relative humidity; 16 L:8 D). A detailed description of the experimental procedure is given by Shiojiri *et al.* [22]. Each wasp was released halfway between the two plants. The first plant visited by the wasp was scored as its choice, then the wasp was removed from the cage. The experiment was conducted during three experimental days (10.00–15.00). The data from these dual-choice tests were analysed using a replicated *G*-test [21]. Wasps that did not make a choice in 30 min were excluded from further statistical analysis.

### 2.5 Predation by ants of two herbivore species

Predation preferences of *L. japonicus*, *Pr. pungens* and *Pa. flavipes* for one larva of *Pi. rapae* versus one of *Pl. xylostella* were assessed in two-choice experiments carried out in the feeder set-up (figure 1*a*,*b*). An aqueous solution of sugar and water in Petri dishes (4 cm in diameter) was supplied. The dish was situated in a climate-controlled room (25±2°C, 50–70% relative humidity; 16 L:8 D). We also scored which of the two larvae was contacted first and predated by an individual ant first. We used second-instar *Pi. rapae* larvae and third-instar *Pl. xylostella* larvae because they were quite similar (5–6 mm) in length. Predation (or contact) scores were then tested against the null hypothesis that the predation events were binomially distributed with equal probability for each of the two species of larvae.

The predation rate of *Pr. pungens* on *Pi. rapae* larvae when offered together with various numbers of *Pl. xylostella* larvae was also examined in the feeder set-up. Five second-instar *Pi. rapae* and 0, 5, 10 or 15 third-instar *Pl. xylostella* were placed in the feeder. Every 15 min, we recorded the number of *Pi. rapae* larvae present in the feeder. Using the same procedure, we also carried out the test with 15 third-instar *Pl. xylostella* and various numbers (0, 5, 10 or 15) of *Pi. rapae* larvae. We repeated each experiment five times, and subjected the data of each observation record to Tukey’s honest significance test (HSD) (*α*=0.05).

Predation by ants on *Pi. rapae* larvae and *Pl. xylostella* larvae was also tested in a cage (180×180×180 cm) in the experimental field of Graduate School of Agriculture, Kyoto University. Inside the cage, there were ant nests (*Pa. flavipes*, *L. japonicus* and *Formica japonica*). We placed a glass Petri dish (10 cm diameter, 1 cm high) containing a cabbage leaf (*ca* 6 cm long), 10 second-instar *Pi. rapae* and 10 third- to fourth-instar *Pl. xylostella* in the experimental field cage covered with gauze (mesh size=1.8 mm). No crucifer plant was grown in the cage. Then, 1.5 and 3 h after the start of the experiment, we placed a small amount of sugar crystals (*ca* 3 g) in the Petri dish, observed the number of surviving larvae of each herbivore species and prevented any other arthropod predator from entering the cage. Each experiment was repeated four times. For each observation time, the numbers of surviving larvae of each herbivore species were subjected to the Wilcoxon–Mann–Whitney test on the differences in survival rates between the two species.

## 3. Results

### 3.1 Oviposition preference of *Pieris rapae*

**Figure 2.**
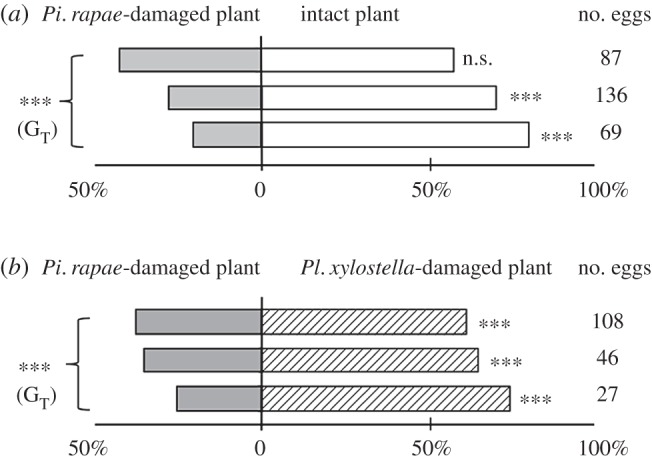
Oviposition preferences of *Pieris rapae* when offered a choice between uninfested plants and *Pi. rapae*-infested plants (*a*) or between *Pi. rapae*-infested plants and *Plutella xylostella*-infested plants (*b*). Significance levels according to *G*-tests are indicated by asterisks; ^**^0.001≤*p*<0.01.

In this study, we asked whether *Pi. rapae* females showed different oviposition preferences for the same plant species with different infestation histories. *Pieris rapae* females significantly preferred uninfested plants to *Pi. rapae*-infested ones (*G*_P_=42.48, d.f.=1, *p*<0.001; *G*_H_=9.27, d.f.=2, *p*=0.01; *G*_T_=51.76, d.f.=3, *p*<0.001; figure 2*a*). The significance of *G*_H_ was due to the fact that the first experiment was not significant (the upper bar of figure 2*a*: *G*=1.95, *p*=0.16), whereas other two experiments were highly significant (the middle bar of figure 2*a*: *G*=23.76, *p*<0.001, the lower bar of figure 2*a*, *G*=26.0, *p*<0.001). Comparison of the attraction of *Pi. rapae* with *Pl. xylostella*-infested plants and *Pi. rapae*-infested plants revealed that *Pi. rapae* preferred the former (*G*_P_=15.75, d.f.=1, *p*<0.001; *G*_H_=1.43, d.f.=2, *p*=0.49; *G*_T_=17.18, d.f.=3, *p*<0.001; figure 2*b*).

### 3.2 Performance of *Pieris rapae* larvae reared on uninfested plants or conspecific-infested plants

The above-described preferences might be explained by the differences in performance of *Pi. rapae* larvae among uninfested, *Pl. xylostella*-infested, and *Pi. rapae*-infested plants. Previously, we reported that the performance of *Pi. rapae* larvae on *Pl. xylostella*-infested plants and on uninfested plants were not significantly different [11]. Here, we compared the performance of *Pi. rapae* larvae on *Pi. rapae*-infested plants and on uninfested plants and found that the number of larvae surviving until pupation on uninfested plants was 16 (*n*=20) and on *Pi. rapae*-infested plants was 18 (*n*=20) and thus not significantly different (Fisher’s exact probability test, d.f.=1, *p*=0.661). Furthermore, no significant difference was observed in pupal weight (*t*-test, *t*_30_=1.15, *p*=0.258) or duration of the larval stage (*t*-test, *t*_30_=1.65; *p*=0.109) between *Pi. rapae* reared on uninfested cabbage plants and on cabbage plants infested by *Pi. rapae* larvae (table 1).

**Table 1.** Performance of *Pieris rapae* larvae reared on uninfested plants or on plants infested by conspecific larvae. (None of the treatments caused significantly different effects.)

plant state	*n*	pupal weight (mg±s.e.)	larval-stage duration (days±s.e.)
uninfested	16	168±26	12.75±0.25
infested by *Pi. rapae* larvae	18	177±15	12.88±0.12

### 3.3 Flight responses of the parasitoid *Cotesia glomerata* to *Pieris rapae*-infested plants

We tested whether specialist parasitoids affect the oviposition preference of *Pi. rapae* females for *Pi. rapae*-infested plants with different levels of damage. *Cotesia glomerata* females showed a significant preference for plants with 30% infestation by *Pi. rapae* larvae (27 individuals) over those with 10% infestation (11 individuals; *G*-test, *G*_t_=6.95, *p*=0.008; heterogeneity among samples: *G*_h_=0.76, *p*=0.38; pooled effect of treatment: *G*_P_=7.71, *p*=0.02). The results suggested that the offspring of a *Pi. rapae* female on a plant already infested by *Pi. rapae* larvae would suffer higher parasitism by *C. glomerata* than *Pi. rapae* larvae on an uninfested plant that had not suffered caterpillar damage by previous infestation of *Pi. rapae* larvae.

### 3.4 Predation by ants of two herbivore species

We then tested how generalist predators affect the oviposition preferences of *Pi. rapae*. Here, we focused on ant species that are major predators of the larvae under field conditions. All three ant species (*L. japonicus*, *Pr. pungens* and *Pa. flavipes*) significantly preferred *Pl. xylostella* larvae to *Pi. rapae* larvae in the two-choice tests (table 2). No significant difference was observed in the first contact of the ants with the herbivores (table 2).

**Table 2.** Predation preferences of three ant species (*Lasius japonicus*, *Pristomyrmex pungens* and *Paratrechina flavipes*) towards *Pieris rapae* larva versus *Plutella xylostella* larva. (*Binomial test.)

	the first contact			the first predation		
	*Pi. rapae*	*Pl. xylostella*	p*	*Pi. rapae*	*Pl. xylostella*	p*
*L. japonicus*	23	30	0.21	30	4	3.08×10^−6^
*Pr. pungens*	11	9	0.75	20	0	9.53×10^−7^
*Pa. flavipes*	23	18	0.83	39	0	1.82×10^−12^

**Figure 3.**
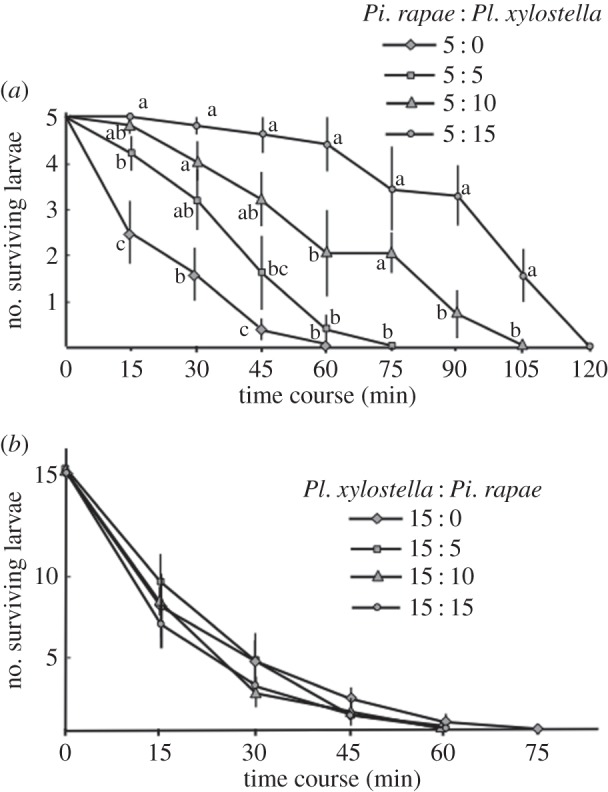
Survival of *Pieris rapae* (*a*) and *Plutella xylostella* (*b*) larvae when feeding together in a cage with an artificial ant nest. Significant differences according to Tukey’s HSD (*p*<0.05) are indicated by different letters.

Next, at different constant supplies of *Pl. xylostella* larvae, predation by *Pr. pungens* on *Pi. rapae* larvae was assessed. When the number of *Pi. rapae* larvae was constant (i.e. five) and the number of *Pl. xylostella* larvae was varied from 0 to 15, predation on *Pi. rapae* larvae significantly decreased with increasing numbers of the latter (figure 3*a*: Tukey’s HSD, *α*=0.05). When the number of *Pl. xylostella* larvae was constant (i.e. 15) and the number of *Pi. rapae* larvae was varied (*n*=0, 5, 10 or 15), there were no significant differences in the predation rate on the former with increasing numbers of the latter (figure 3*b*: Tukey’s HSD, *α*=0.05).

**Figure 4.**
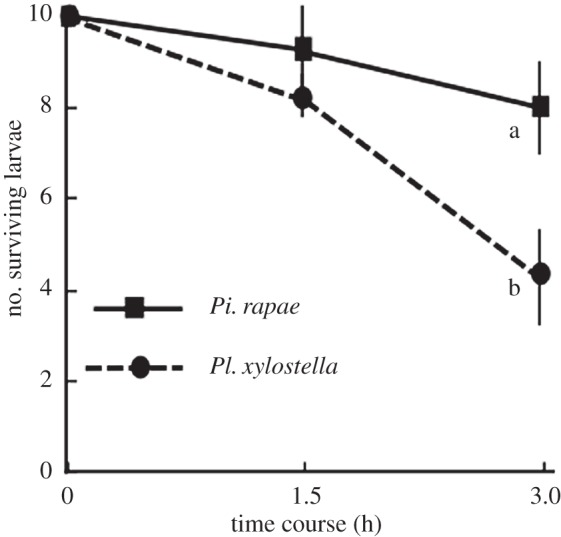
Effect of *Pieris rapae* and *Plutella xylostella* larvae together (i.e. inhabiting the same place in the field) on their respective survival rates. Significant differences according to the Mann–Whitney *U*-test (*p*<0.05) are indicated by different letters.

The predation rates by ants on *Pi. rapae* and *Pl. xylostella* larvae were also assessed in the field. The ant species observed in the study field were *Pa. flavipes*, *L. japonicus* and *Formica japonica*. At 1.5 h after the start of the experiment, no significant difference was observed in the number of surviving larvae between *Pi. rapae* and *Pl. xylostella* (Wilcoxon–Mann–Whitney test: *W*=8.5; *p*=0.45). However, after 3 h only 42.5% of *Pl. xylostella* larvae survived, whereas 80% of *Pi. rapae* larvae survived (Wilcoxon–Mann–Whitney test: *W*=1; *p*=0.020; figure 4).

## 4. Discussion

No significant difference was observed in the performance (larval stage duration or pupal weight) of *Pi. rapae* larvae reared on the two types of cabbage plants (i.e. uninfested plants and plants infested by *Pi. rapae* larvae; table 1). Further, we previously reported that no significant differences in the performance were observed between *Pi. rapae* larvae reared on *Pl. xylostella*-infested plants and those reared on uninfested plants [11]. Thus, in this study, we concluded that the observed preferences by *Pi. rapae* females did not occur because of the induction of chemical defence by either *Pi. rapae* larvae or *Pl. xylostella* larvae; that is, it is unlikely the presence of these larvae made uninfested plants a more profitable food source. Thus, the preference-performance hypothesis can be ruled out in the preferences in figure 2. We discussed the results from the viewpoints of: (i) resource competition, (ii) intraguild predation, (iii) herbivore-induced enemy-rare/dense space, and (v) apparent interaction.

### 4.1 Uninfested plants versus plants infested by *Pieris rapae* larvae

*Pieris rapae* females preferred uninfested cabbage plants over those infested by conspecific larvae (figure 2*a*). Sato *et al*. [18] observed a similar oviposition preference of *Pi. rapae* females for uninfested wild *Rorippa indica* (Brassicaceae). Intraspecific resource competition may explain the preference for uninfested plants. Another possible explanation is that avoiding plants with conspecific larvae prevents the eggs being cannibalized by conspecific larvae (intraspecific predation) [23].

In addition to these direct effects, indirect effects may also explain the preference of *Pi. rapae* females for uninfested plants. The parasitism rate of *Pi. rapae* larvae by *C. glomerata* can sometimes be considerable; it varies in time and may occasionally exceed 70% [24]. We previously reported that *C. glomerata* preferred plants infested by *Pi. rapae* larvae, its host, to uninfested plants [22], and we showed here that cabbage plants become more attractive to *C. glomerata* with increasing damage by host larvae. Thus, when eggs are deposited on cabbage plants infested by conspecifics, the emerging larvae may face an occasionally higher risk of *C. glomerata* parasitism than those hatched from eggs deposited on uninfested plants.

### 4.2 Plants infested by *Plutella xylostella* versus *Pieris rapae*

We found that *Pi. rapae* females preferred *Pl. xylostella*-infested to *Pi. rapae*-infested plants (figure 2*b*). In this preference, what matters to *Pi. rapae* females is not how much competition their larvae will experience immediately on the plant but rather how much they will suffer from predation before reaching maturity. Interspecific differences in the rate at which plant damage accumulates during larval development are important and should be taken into account. In this respect, the two herbivore species were extremely dissimilar; the total amount of damage done by one *Pi. rapae* larva before pupation to yellow cress plants (*R. indica*) [25] is *ca* 25 times higher than that by one *Pl. xylostella* larva to cabbage seedlings [26]. Thus, *Pi. rapae*-infested plants would incur more total damage than *Pl. xylostella*-infested ones (intraspecific resource competition). The assumption that *Pi. rapae* females do not select oviposition sites simply based on the leaf area presently available as a resource is also supported by the fact that these females preferred artificially damaged wild *R. indica* to those that received the same amount of damage caused by feeding of conspecific larvae [18].

The amount of damage to a plant is positively correlated with its attractiveness to the parasitoid *C. glomerata*, irrespective of the parasitoid’s target herbivore species (this study and [22,27]). Thus, the offspring of *Pi. rapae* females would suffer higher parasitism when *Pi. rapae* eggs were deposited on conspecific-infested plants than when they were deposited on *Pl. xylostella*-infested plants because of the difference in net damage accumulation between the two herbivore species (i.e. higher cost of herbivore-induced enemy-dense space).

To further determine why *Pi. rapae* females preferred *Pl. xylostella* larvae-infested plants to *Pi. rapae* larvae-infested plants, we studied the roles of generalist predators (ants) shared by the two herbivore species. Direct observation showed that ants of three species preferred to prey on *Pl. xylostella* larvae first (table 2). Furthermore, predation by ants on *Pi. rapae* larvae gradually decreased with an increasing number of available *Pl. xylostella* larvae (figure 3*a*). This reduced predation by ants on *Pi. rapae* larvae could not simply be based on an increase in the total number of larvae, because adding more *Pi. rapae* larvae did not affect the risk of predation on *Pl. xylostella*(figure 3*b*). We therefore hypothesized that ants preferred *Pl. xylostella* larvae to *Pi. rapae* larvae. In the field experiments (figure 4), ants fed exclusively on *Pl. xylostella* larvae when both species were present, supporting the above hypothesis. This observed preference of ants for *Pl. xylostella* larvae is thought to be the result of the presence of oily drops on the dorsal setae of *Pi. rapae* larvae [28], which are thought to act as a defence against ants [29,30]. Because *Pl. xylostella* larvae lack such a defence system of oily drops on top of the dorsal setae (Shiojiri and Takabayashi, direct observation), ants prefer *Pl. xylostella* larvae when both herbivore species are present on the same plant (assuming all else being equal). In the laboratory experiment, all larvae were consumed by the ants within 2 h (figure 3), whereas the rate of predation on each of the two herbivore species was lower in the field experiments: slightly more than half of the *Pl. xylostella* larvae had been consumed by ants in 3 h, whereas most *Pi. rapae* larvae were still alive (figure 4). This probably occurred, because: (i) in the artificial ant nests, *Pi. rapae* and *Pl. xylostella* larvae were the only available protein sources, and (ii) the distance between prey and any ant nest was larger in the field than in the laboratory. Therefore, the predation pressure by ants on *Pi. rapae* larvae would be substantially reduced when they occur together with *Pl. xylostella* larvae on the same plants under natural conditions (apparent competition). All these factors would explain why females of *Pi. rapae* deposited fewer eggs on conspecific-infested plants than on *Pl. xylostella*-infested plants.

### 4.3 Uninfested plants versus plants infested by *Plutella xylostella* larvae

We previously reported that *Pi. rapae* females showed no preference when offered *Pl. xylostella*-infested plants versus uninfested plants [11]. Clearly, *Pi. rapae* and *Pl. xylostella* larvae compete for the same resources. Intraguild predation was unlikely, because *Pl. xylostella* larvae do not prey on *Pi. rapae* eggs (K. Shiojiri 1999, unpublished data). For indirect effects, *Pl. xylostella*-infested plants attracted *C. glomerata* to the same degree as *Pi. rapae*-infested plants [22]; therefore, the oviposition on *Pl. xylostella*-infested plants would result in a higher incidence of parasitism (herbivore-induced enemy-dense space). All these data suggested that *Pi. rapae* females preferred uninfested plants to those infested by *Pl. xylostella*, but the observed results were opposite [11]. Here, we partly explained this paradox by showing the fact that there was a net benefit from associating with *Pl. xylostella,* because of the positive effect of apparent competition through generalist predators (i.e. ants) that might compensate for the resource competition and apparent competition mediated by specialist predators, such as *C. glomerata*.

### 4.4 A perspective on interspecific herbivore associations

Newly emerged *Pi. rapae* females always disperse from their natural habitat, even if that habitat still contains enough food for their offspring [10,31]. This dispersal behaviour may increase the likelihood of *Pi. rapae* females finding habitats in which *C. glomerata*, a specialist parasitoid of their larvae, is absent or rare [24]. In such a new habitat, other herbivores (e.g. *Pl. xylostella* larvae) and other—usually generalist—predators (e.g. ants) may prevail. Consequently, the first colonizers of this new habitat need to cope only with the risk of being eaten by generalist predators, and to compete with other herbivores on the same plants. By contrast, females of *Pi. rapae* that arrive later are also threatened by the specialist parasitoid *C. glomerata* that will follow the invaders, albeit with a delay. Furthermore, females of *Pl. xylostella* preferred to lay eggs (future competitors) on plants already infested with *Pi. rapae* larvae [11].

Under these conditions, *Pl. xylostella* and *Pi. rapae* larvae probably co-occur on specific plants, and in this situation, both are threatened by their respective specialist parasitoids and generalist predators, ants. When co-occurring, the presence of *Pl. xylostella* larvae increases the incidence of parasitism of *Pi. rapae* larvae by *C. glomerata*, because the plant volatiles become more attractive to the parasitoid, but the rate of predation by ants declines (figure 4*a*). By contrast, the coexistence of *Pi. rapae* and *Pl. xylostella* larvae on a plant results in a decreased incidence of parasitism of *Pl. xylostella* by *Cotesia plutellae*, a specialist parasitic wasp of *Pl. xylostella* larvae [11], but effects of such coexistence on the ant predation pressure on *Pl. xylostella* larvae would not be changed (figure 4*b*).

We studied here the oviposition preferences of a small white butterfly, *Pi. rapae*, for plants occupied by combinations of conspecific larvae, heterospecific larvae (*Pl. xylostella*), specialist parasitoid *C. glomerata* and generalist predators (ants). As is illustrated by our case study, the value of a host plant to a female herbivore depends on trophic interactions (preference-performance hypothesis, herbivore-induced enemy-rare/dense space and apparent competition), as well as on non-trophic interactions (inter- and intraspecific resource competition and intraguild predation). Recently, it has been shown that egg deposition stimuli change the physiology of plants and subsequently the performance of hatched larvae and their natural enemies [32–35]. Such aspects should also be tested regarding the oviposition preferences of female herbivores. The oviposition preferences of herbivorous insects can only be explained by integrating these trophic and non-trophic interactions, and clearly, this line of argument can be extended from host/food plant selection by herbivores to the development of community structures of arthropods on plants.

## Data accessibility

Supporting data available at http://dx.doi.org/10.5061/dryad.91bj3.
